# Complex Chromatin Motions for DNA Repair

**DOI:** 10.3389/fgene.2020.00800

**Published:** 2020-08-27

**Authors:** Judith Miné-Hattab, Irene Chiolo

**Affiliations:** ^1^UMR 3664, CNRS, Institut Curie, PSL Research University, Paris, France; ^2^UMR 3664, CNRS, Institut Curie, Sorbonne Université, Paris, France; ^3^Molecular and Computational Biology Department, University of Southern California, Los Angeles, CA, United States

**Keywords:** chromatin motions, double-strand break repair, homologous recombination, mean square displacement, directed motion, multi-scale motion

## Abstract

A number of studies across different model systems revealed that chromatin undergoes significant changes in dynamics in response to DNA damage. These include local motion changes at damage sites, increased nuclear exploration of both damaged and undamaged loci, and directed motions to new nuclear locations associated with certain repair pathways. These studies also revealed the need for new analytical methods to identify directed motions in a context of mixed trajectories, and the importance of investigating nuclear dynamics over different time scales to identify diffusion regimes. Here we provide an overview of the current understanding of this field, including imaging and analytical methods developed to investigate nuclear dynamics in different contexts. These dynamics are essential for genome integrity. Identifying the molecular mechanisms responsible for these movements is key to understanding how their misregulation contributes to cancer and other genome instability disorders.

## Introduction: Chromatin Explores a Larger Nuclear Volume in Response to DNA Damage

**FIGURE 1:**
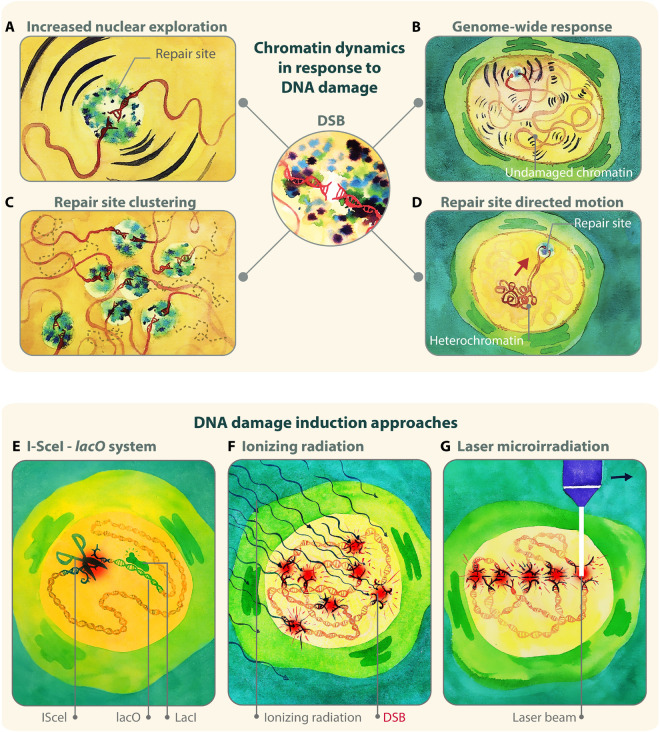
Examples of damage-induced changes in chromatin dynamics. **(A)** A damaged chromatin site explores a larger nuclear volume during HR repair of DSBs. **(B)** Larger nuclear exploration is also observed for undamaged chromatin sites, indicating that the change in chromatin mobility is a genome-wide response. **(C)** Multiple DSB repair sites cluster together. **(D)** Damaged heterochromatic sites relocalize to the nuclear periphery with directed motions in Drosophila cells. **(E,F)** Three main approaches to study nuclear dynamics in response to DSBs rely on: **(E)** enzymatic digestion to induce damage and lacO or tetO arrays to follow damage sites; **(F)** IR to induce damage and fluorescent tagging of repair proteins to detect repair foci; **(G)** laser, alpha particles, or heavy ions, to induce damage along linear tracks where repair protein recruitment is detectable by live imaging or immunofluorescence. Illustration by Olga Markova.

A significant number of studies in the past decade have identified essential roles for nuclear dynamics in DNA repair, particularly during homologous recombination (HR) repair of double-strand breaks (DSBs) (Figures 1A–D). First, a larger nuclear volume explored by repair sites is typically detected during inter-homolog recombination (Figure 1A) (Miné-Hattab and Rothstein, 2012; Neumann et al., 2012; Cho et al., 2014; Miné-Hattab et al., 2017) (reviewed in Dion and Gasser, 2013; Mine-Hattab and Rothstein, 2013). This change in chromatin mobility in response to DNA damage likely reflects the exploration of the nuclear space during “homology search” (Kalocsay et al., 2009; Dion et al., 2012; Miné-Hattab and Rothstein, 2012; Neumann et al., 2012; Agmon et al., 2013; Cho et al., 2014; Saad et al., 2014; Herbert et al., 2017; Miné-Hattab et al., 2017), i.e., the process where a resected DSB covered by a Rad51 nucleoprotein filament scans the genome in search of a homologous donor. Second, undamaged chromatin also becomes more dynamic during DSB repair, albeit to a lesser extent than repair sites (Figure 1B) (Chiolo et al., 2011; Krawczyk et al., 2012; Miné-Hattab and Rothstein, 2012; Seeber et al., 2013; Lottersberger et al., 2015; Strecker et al., 2016; Herbert et al., 2017; Lawrimore et al., 2017; Miné-Hattab et al., 2017; Caridi et al., 2018a; Smith et al., 2019; Zada et al., 2019). The significance of the genome-wide increase in nuclear exploration is still under debate, but this response might increase the frequency of DNA contacts to facilitate homology search (Gehen et al., 2011; Neumann et al., 2012; Mine-Hattab and Rothstein, 2013; Amitai and Holcman, 2018), or reflect chromatin relaxation to promote access for repair (Kruhlak et al., 2006; Ziv et al., 2006; Seeber et al., 2013; Delabaere and Chiolo, 2016). Third, repair sites undergoing HR aggregate into larger units, or “clusters” (Figure 1C) (Lisby et al., 2003; Aten et al., 2004; Kruhlak et al., 2006; Chiolo et al., 2011, 2013; Krawczyk et al., 2012; Neumaier et al., 2012; Cho et al., 2014; Caron et al., 2015; Aymard et al., 2017; Caridi et al., 2018a; Schrank et al., 2018; Oshidari et al., 2019a; Waterman et al., 2019) (reviewed in Chiolo et al., 2013; Guénolé and Legube, 2017; Schrank and Gautier, 2019), likely to facilitate DSB signaling and resection, e.g., by increasing the local concentration of checkpoint and repair proteins (Chiolo et al., 2013; Schrank et al., 2018; Kilic et al., 2019; Oshidari et al., 2019a; Schrank and Gautier, 2019). This response also occurs in G1 in human cells (Aten et al., 2004; Aymard et al., 2017), where HR cannot be completed with the sister chromatid, and clustering might temporarily sequester breaks that will be repaired in S phase (Aymard et al., 2017). Clustering can also be deleterious, as increasing the proximity of DSBs on different chromosomes promotes chromosomal translocations (Agmon et al., 2013; Roukos et al., 2013; Lee et al., 2016; Cohen et al., 2018). Fourth, DSBs relocalize to specific subnuclear compartments when the lesion occurs in DNA regions that are difficult to repair. Specifically, DSBs in pericentromeric heterochromatin relocalize to the nuclear periphery in *Drosophila* cells (Figure 1D) (Chiolo et al., 2011; Ryu et al., 2015, 2016; Janssen et al., 2016, 2019; Caridi et al., 2018a), and to the periphery of heterochromatin “domains” (or “chromocenters”) in mouse cells (Jakob et al., 2011; Chiolo et al., 2013; Tsouroula et al., 2016; Caridi et al., 2018a). rDNA sequences move to the nuclear periphery in budding yeast (Torres-Rosell et al., 2007; Horigome et al., 2019) and to nucleolar caps in mammalian cells (Harding et al., 2015; van Sluis and McStay, 2015; Korsholm et al., 2019; Marnef et al., 2019). Relocalization of repair sites to the nuclear periphery is also a response to damaged CAG repeats in budding yeast (Su et al., 2015; Aguilera et al., 2020; Whalen et al., 2020), collapsed replication forks in yeast and mammalian cells (Nagai et al., 2008; Su et al., 2015; Lamm et al., 2018; Aguilera et al., 2020; Whalen et al., 2020), and damaged telomeric or subtelomeric sequences in yeast (Therizols et al., 2006; Khadaroo et al., 2009; Cho et al., 2014; Chung et al., 2015; Churikov et al., 2016; Oshidari et al., 2018; Aguilera et al., 2020). Similar relocalization occurs as a result of persistent/unrepairable DSBs (Nagai et al., 2008; Kalocsay et al., 2009; Oza et al., 2009; Horigome et al., 2014, 2016; Swartz et al., 2014; Marcomini et al., 2018). In these contexts, relocalization appears to prevent aberrant recombination with ectopic repeated sequences (Torres-Rosell et al., 2007; Chiolo et al., 2011; Ryu et al., 2015, 2016; Su et al., 2015; Caridi et al., 2018a; Dialynas et al., 2019; Aguilera et al., 2020) and/or promote alternative repair mechanisms (Therizols et al., 2006; Nagai et al., 2008; Khadaroo et al., 2009; Oza et al., 2009; Horigome et al., 2014, 2016; Churikov et al., 2016; Aguilera et al., 2020) (reviewed in Amaral et al., 2017; Caridi et al., 2017, 2019; Rawal et al., 2019). Further dynamics have been associated with other repair pathways. For example, deprotected telomeres are mobilized in mouse cells to promote non-homologous end-joining (NHEJ) (Dimitrova et al., 2008; Lottersberger et al., 2015). Additionally, a few chromosome territories reposition in response to damage in human fibroblasts, perhaps reflecting large-scale changes in chromatin organization (Mehta et al., 2010; Kulashreshtha et al., 2016). Several molecular mechanisms governing chromatin dynamics in response to DSBs have been identified, and specialized pathways appear to participate in different contexts [reviewed in Amaral et al. (2017); Caridi et al. (2017); Zimmer (2018); Oshidari et al. (2019b)]. Together, these studies revealed important roles for nuclear dynamics in DSB repair, particularly for homology search and for isolating repeated DNA sequences at high risk for aberrant recombination, enabling “safe” repair or alternative rescue pathways.

## Methods to Characterize Nuclear Dynamics During DSB Repair

Several techniques have been applied to study the nuclear dynamics of DSBs in different organisms, with “gold standard” approaches relying on damage induction with endonucleases or ionizing radiation (IR), and on monitoring repair sites with *lacO/tetO* arrays and fluorescent-tagged HR repair components (Figures 1E–G).

A widely used approach relies on the induction of site-specific DSBs with an endonuclease (e.g., I-*Sce*I, or HO), which recognizes a target sequence proximal to *tetO* or *lacO* arrays (Figure 1E). The position of the damage site is monitored using fluorescent-tagged TetR or LacI proteins that bind to the arrays, resulting in bright nuclear spots (or *foci*) (Robinett et al., 1996). Given that endonucleases can digest both sister chromatids, the sister is mostly unavailable as a template for repair, promoting inter-homolog exchanges (Miné-Hattab and Rothstein, 2012). In yeast, this approach enabled the study of nuclear dynamics associated with inter-homologous recombination (Miné-Hattab and Rothstein, 2012; Neumann et al., 2012; Miné-Hattab et al., 2017), unrepairable DSBs (Nagai et al., 2008; Kalocsay et al., 2009; Dion et al., 2012; Horigome et al., 2014, 2016; Saad et al., 2014; Herbert et al., 2017), sub-telomeric breaks (Khadaroo et al., 2009; Chung et al., 2015; Churikov et al., 2016; Oshidari et al., 2019b), and rDNA lesions (Torres-Rosell et al., 2005). Similar systems have been used to characterize DSB clustering in budding yeast and human cells (Lisby et al., 2003; Roukos et al., 2013; Waterman et al., 2019). A variant of this approach employs a *lacO* array close to the cut site and a *tetO* array on a different chromosome, enabling the simultaneous tracking of both damaged and undamaged loci (Miné-Hattab and Rothstein, 2012; Miné-Hattab et al., 2017). These studies and others (Seeber et al., 2013; Herbert et al., 2017) revealed that not only damaged sites, but also undamaged chromatin explores a larger nuclear volume in response to DSB formation. An alternative system employed sequence-based tethering of oligomerizing fluorescent proteins that spread along the DNA, to study the dynamics of resected DNA (Saad et al., 2014). Here, resection results in loss of DNA-associated proteins and reduced signal at repair sites, and correlates with a local reduction in focus dynamics (Saad et al., 2014). These approaches are very powerful, but also limited to the site targeted by the endonuclease. Given that repair responses and relocalization pathways are affected by chromatin state (Delabaere and Chiolo, 2016; Hauer and Gasser, 2017), nuclear positioning (Lemaitre et al., 2014), and the phase separated nature of nuclear subdomains (Altmeyer et al., 2015; Kilic et al., 2019; Lenzken et al., 2019; Oshidari et al., 2019a; Pessina et al., 2019) (reviewed in Clouaire and Legube, 2019; Rawal et al., 2019), the endonuclease-based approach would need to be applied to a large number of sites to provide a comprehensive understanding of the mechanisms responsible for these dynamics in different contexts.

Site-specific endonucleases have also been used to induce DSBs in highly repeated DNA sequences. For example, studies using Cas9 targeting heterochromatic satellites (Tsouroula et al., 2016) or PpoI or Cas9 recognizing rDNA sequences (Harding et al., 2015; van Sluis and McStay, 2015; Korsholm et al., 2019; Marnef et al., 2019) revealed the dynamics of these sites in mammalian cells. It is important to consider that Cas9 affects the processing of repair intermediates (Richardson et al., 2016, 2018; Brinkman et al., 2018), potentially affecting outcomes and dynamics of repair.

Another commonly used approach relies on inducing damage with ionizing radiation (IR), and detecting repair sites using fluorescent-tagged HR components (Figure 1F). This has been an invaluable approach to characterize focus mobility relative to other nuclear structures, such as the heterochromatin domain, the nuclear periphery, or other repair foci (Kruhlak et al., 2006; Falk et al., 2007; Miné-Hattab and Rothstein, 2012; Chiolo et al., 2013; Lottersberger et al., 2015; Ryu et al., 2015, 2016; Caridi et al., 2018a, b; Schrank et al., 2018; Dialynas et al., 2019; Oshidari et al., 2019a; See et al., 2020). These studies established, for example, that heterochromatic DSBs move to the nuclear periphery to continue repair in *Drosophila* cells (Chiolo et al., 2011, 2013; Ryu et al., 2015, 2016; Caridi et al., 2018a). A major advantage of inducing DSBs with IR, relative to using chemical treatments or enzymatic digestion, is that IR-induced DSBs form within a very tight time window. This enables synchronous responses, and an easier characterization of focus dynamics and kinetics at the population level, including in animal tissues (see for example: Lisby et al., 2004; Delabaere et al., 2017; Caridi et al., 2018b; See et al., 2020). Further, IR treatments are well suited to damaging chromatin that is difficult to access with enzymatic digestion, such as heterochromatin (Goodarzi et al., 2008; Iacovoni et al., 2010; Chiolo et al., 2011; Ryu et al., 2015, 2016; Caridi et al., 2018a, b). A potential limitation of this approach is that tracking several sites in the nuclei requires frequent time points to minimize ambiguous tracks, which increases the potential for photobleaching and phototoxicity effects in long kinetics (Caridi et al., 2018b; See et al., 2020).

Alternative approaches employed lasers, alpha-particles, or heavy ions to induce damage along linear tracks in the nucleus of mammalian cells (Figure 1G), and repositioning of damage sites is monitored relative to these tracks and specific nuclear compartments (Aten et al., 2004; Chiolo et al., 2011; Jakob et al., 2011; Reynolds et al., 2013). These approaches revealed, for example, that repair foci cluster over time (Aten et al., 2004), and that damage in pericentric heterochromatin results in relocalization of repair sites to outside the chromocenters in mouse cells (Jakob et al., 2011). Using laser beams mounted on a microscope is also an effective method to investigate early damage responses (Bekker-Jensen et al., 2006). However, the high energy associated with some of these damage sources might also impair the chromatin state and repair outcomes (Singleton et al., 2002; Lukas et al., 2005; Reynolds et al., 2013; Kong et al., 2018), and even directly affect relocalization mechanisms (Chiolo et al., 2011).

Thus, several approaches have been developed to characterize the dynamics of repair foci. The preferred method depends on the type of question and the features of the motion under investigation.

## MSD Analysis

**FIGURE 2:**
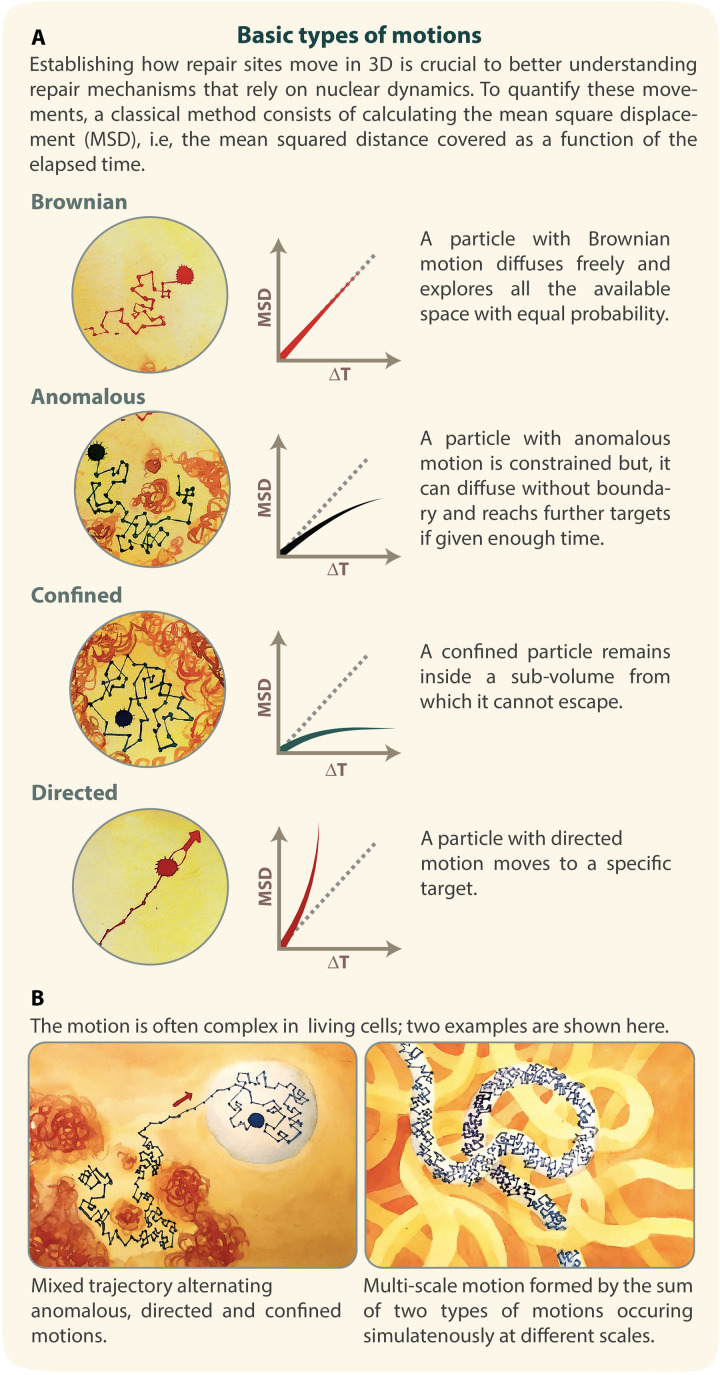
MSD curves identify different types of motions. **(A)** Illustration of Brownian, anomalous, confined and directed motions, with corresponding MSD curves. **(B)** Examples of complex motion. Left: a mixed trajectory alternating anomalous, directed, and confined motion. Right: a motion of a site characterized by a diffusion coefficient Amicro, in a region that itself diffuses with a diffusion coefficient Amacro. Inspired by De Gennes’s reptation model (Gennes, 1982). Illustration by Olga Markova.

A traditional approach to characterize the dynamics of damaged DNA is the mean-square displacement (MSD) analysis of the positional data of repair sites (reviewed in Spichal and Fabre, 2017; Caridi et al., 2018b). The MSD curve represents the amount of space a locus explores in the nucleus, and its shape has been used to describe the nature of the movement (Michalet and Berglund, 2012; Oswald et al., 2014; Spichal and Fabre, 2017; Caridi et al., 2018b; Figure 2). The time-averaged MSD of a single trajectory is calculated using the following equation:

$$\text{MSD}\left(n\cdot \Delta t\right)=\frac{1}{N-n}\sum_{i=1}^{N-n}\left[{\left(x_{i+n}-x_{i}\right)}^{2}+{\left(y_{i+n}-y_{i}\right)}^{2}+{\left(z_{i+n}-z_{i}\right)}^{2}\right]$$

where *N* is the number of points in the trajectory, (*x*, *y*, *z*) the coordinates of the locus in 3-dimensions, and Δ*t* the time interval of the acquisition. MSDs are typically calculated for several trajectories across distinct nuclei, and averaged to extract a time-ensemble-averaged MSD. The data are then fitted to a curve to characterize the type of diffusion. In the following sections, we present different models used in the literature to fit averaged MSD curves.

### Brownian Motion

When a molecule freely diffuses, its MSD curve is linear at increased time intervals and its motion is called Brownian (Figure 2A). In this case, the MSD follows:

MSD(Δt)=2dDΔt

where *d* is the dimension of the movement, *D* is the diffusion coefficient of the locus, and Δ*t* is the time interval. The term 2dDΔ*t* is the theoretical MSD for Brownian motion in the absence of any experimental noise. However, when measuring the position of a molecule in living cells, the experimental location accuracy can strongly affect the experimental MSD. The error in location for molecules detected by live imaging can be divided into two components (Supplementary Table S1):

• Error in the determination of the position due to convolution of the sample with the point spread function (PSF). This depends on imaging conditions and microscope features (e.g., the numerical aperture of the objective, the number of photons collected by the camera, and the wavelength of light). This error is higher at short acquisition times since the number of photons collected is small.
• Error due to the movement of the spot during the acquisition. This error, referred as “motion blur” is higher at longer exposure times.

Experimental MSD curves for Brownian motion, taking into account the location errors, can be fitted by Michalet (2010):

$$\text{MSD}\left(\Delta t\right)=2dD\Delta t+\sigma_{0}^{2}\left(1+\frac{Dt_{\text{Exp}}}{s_{0}^{2}}\right)-\frac{4}{3}t_{\text{Exp}}$$

where $\sigma_{0}^{2}$ is the localization accuracy of an immobile particle;

$s_{0}^{2}$ is the variance of the PSF;

*t*_Exp_ is the exposure time of the camera.

### Sub-diffusive Motion

In living cells, DNA motion is often slower than Brownian and is called “sub-diffusive” (Barkai et al., 2012). This is due to the existence of constraints that limit chromatin movement, including the polymeric nature of the chromatin, chromatin compaction, molecular crowding, phase separation, and anchoring to subnuclear compartments (Marshall et al., 1997; Spichal and Fabre, 2017; Caridi et al., 2018b). Two types of sub-diffusive motions have been described: confined sub-diffusion and anomalous sub-diffusion.

#### Confined Sub-diffusion

When a chromosomal locus stays confined inside a sub-volume of the nucleus, its motion is called confined sub-diffusion (Figure 2A). The MSD exhibits a plateau (Marshall et al., 1997) and follows the equation:

$$\text{MSD}\left(\Delta t\right)=R_{\infty }^{2}\left(1-e^{-2dD\Delta t/R_{\infty }^{2}}\right)+\varepsilon$$

where *R*_*∞*_ is the measured plateau of the MSD, *D* is the diffusion coefficient of the locus and ε is the noise due to the experimental measurements. The confinement radius (*Rc*) of the motion is given by the relation: $R_{c}=R_{\infty }\sqrt{\left(d+2\right)/d}$, where *d* is the dimension of the motion. It refers to the radius of a sphere inside which the motion is contained. The MSD curve starts to bend at time $t_{c}=R_{c}^{2}/\left(2dD\right)$, representing the characteristic equilibration time after which the effect of boundaries appears.

#### Anomalous Sub-diffusion

When the force or structure that restricts the motion is not a simple confinement but is modulated in time and space with scaling properties, the motion is called anomalous sub-diffusion (Barkai et al., 2012; Metzler et al., 2014) (Figure 2A). In this case, sub-diffusive loci are constrained, but, unlike confined loci, they can diffuse without boundary and thus reach further targets if given enough time. For sub-diffusive motion, the MSD exhibits a power law,

$$\text{MSD}\left(\Delta t\right)=A\Delta t^{\alpha }+\varepsilon$$

where α, the anomalous exponent, is smaller than 1.

The anomalous exponent α is linked to the degree of recurrence of DNA exploration, i.e., the number of times a locus reiteratively scans neighboring regions before reaching a distant position (Ben-Avraham, 2000). When α is small, the locus recurrently explores the same environment for a long time, while a large α indicates that the locus is able to explore new environments often. The anomalous diffusion coefficient *A* represents the amplitude of the motion; it is proportional to the diffusion coefficient only in the case of normal diffusion (when α = 1), which is rarely observed in biological systems (Barkai et al., 2012).

Experimental noise ε can strongly affect MSD measurements also in the case of anomalous sub-diffusion. The exact formula to fit the MSD curves of anomalous diffusion, including localization accuracy, is given by the formula:

$$\text{MSD}\left(\Delta t\right)=A\Delta t^{\alpha }+\sigma_{0}^{2}\left(1+\frac{At_{Exp}^{\alpha }}{4s_{0}^{2}}\right)-\frac{A\alpha \left(1-\alpha \right)t_{Exp}^{2}}{12\Delta t^{2}}\Delta t^{\alpha }-\frac{2A}{\left(\alpha +1\right)\left(\alpha +2\right)}$$

which has been calculated and used to characterize chromatin mobility at multiple time scales (Miné-Hattab et al., 2017).

### Directed Motion

Recent studies of chromatin mobility in the context of DNA repair have revealed the existence of transient directed motion in living cells (Cho et al., 2014; Caridi et al., 2018a, b; Lamm et al., 2018; Oshidari et al., 2018) (Figure 2A). For directed motion, MSD values rapidly increase at higher time intervals, as follows:

$$\text{MSD}\left(\Delta t\right)=2dD\Delta t+\nu^{2}\Delta t^{2}+\varepsilon$$

where *D* is the diffusion coefficient, ν is the velocity of the directed motion and ε is the noise due to the experimental measurements.

## MSD Analyses Reveal Increased Nuclear Exploration of Damaged and Undamaged Chromatin in Response to DSBs

MSD analyses have been used to characterize the dynamics of repair sites in different contexts, from yeast to mammalian cells, deriving descriptive parameters like diffusion coefficient and confinement radius. In yeast, for example, MSD analyses of repair sites in response to *ISceI*-induced breaks revealed that resected DSBs explore a nuclear volume up to ten times larger than before damage (Dion et al., 2012; Miné-Hattab and Rothstein, 2012). This response depends on resection, chromatin remodeling, checkpoint activation, the strand invasion component Rad51 (Oza et al., 2009; Dion et al., 2012; Miné-Hattab and Rothstein, 2012; Neumann et al., 2012; Horigome et al., 2014; Saad et al., 2014; Amitai et al., 2017; Miné-Hattab et al., 2017; Smith et al., 2018), and it has been linked to homology search (Dion et al., 2012; Miné-Hattab and Rothstein, 2012; Neumann et al., 2012; Miné-Hattab et al., 2017). This process is exceptionally efficient. For example, in *S. cerevisiae*, a single recipient locus and a single donor locus that share as little as 1.2 kb of homology will find each other in the 15,000 kb of genome, and engage in repair with 90% efficiency within 2 h after DSB formation (Aylon et al., 2003; Miné-Hattab and Rothstein, 2012). Increased nuclear exploration is more pronounced in diploid than in haploid cells (Dion et al., 2012; Miné-Hattab and Rothstein, 2012), potentially reflecting a more active search when the homologous partner is available (Mine-Hattab and Rothstein, 2013). Indeed, HR repair with the sister chromatid, which is kept in close proximity through cohesion, is not associated with extensive dynamics (Dion et al., 2012, 2013), further linking nuclear exploration with inter-homologous repair in yeast.

Importantly, studies in yeast revealed that undamaged loci also become more dynamic in response to damage, exploring a nuclear volume up to four times larger, and more DSBs induce larger nuclear exploration (Miné-Hattab and Rothstein, 2012; Seeber et al., 2013; Herbert et al., 2017; Lawrimore et al., 2017; Miné-Hattab et al., 2017). Changes in chromatin mobility are thus a general feature of the cellular response to DSBs affecting the whole genome. Experimental and theoretical studies suggest that changes in chromatin mobility of both damaged and undamaged loci increase the probability of contact between distant loci, thus promoting the kinetics of homologous pairing (Miné-Hattab and Rothstein, 2012; Guerin et al., 2016; Miné-Hattab et al., 2017; Amitai and Holcman, 2018).

Increased nuclear exploration of damaged sites during HR repair is also observed in mammalian and *Drosophila* cells (Chiolo et al., 2011; Krawczyk et al., 2012; Becker et al., 2014; Cho et al., 2014; Lottersberger et al., 2015; Ryu et al., 2015; Caridi et al., 2018a, b; Schrank et al., 2018). Studies of Rad52 foci in S phase of human cells revealed significant dynamics even when the sister chromatid is used as a template, and linked it to clustering of repair sites (Schrank et al., 2018) (Supplementary Table S1). Notably, in human cells NHEJ appears to operate more frequently than HR (Beucher et al., 2009), and does not require extensive movement (Krawczyk et al., 2012; Aymard et al., 2017; Schrank et al., 2018; Schrank and Gautier, 2019), except at unprotected telomeres (Dimitrova et al., 2008; Lottersberger et al., 2015). This might explain why repair focus dynamics have not been detected in early studies (Nelms et al., 1998; Soutoglou et al., 2007; Jakob et al., 2009). Further, studies in *Drosophila* cells treated with IR, revealed that both euchromatic and heterochromatic repair foci are mobilized (Caridi et al., 2018a), with the most extensive nuclear exploration associated with heterochromatic sites that relocalize to the nuclear periphery (Ryu et al., 2015; Caridi et al., 2018a).

Although the movement of undamaged sites has not been consistently tracked in these systems, the dynamics of other (undamaged) chromosomal loci (e.g., telomeres and centromeres) before and after damage suggest that global chromatin mobilization is also conserved (Lottersberger et al., 2015; Caridi et al., 2018a).

What promotes the dynamics of undamaged loci in response to DSBs? Different contributing mechanisms have been identified: (i) the release of structures that anchor chromosomal loci to the nuclear periphery, (ii) repair and checkpoint proteins; (iii) the transfer of cytoplasmic forces to the chromatin through the LINC complex; and (iv) global chromatin modifications. Specifically, anchoring of centromeres, telomeres and the nucleolus to the nuclear envelope provides constraints to the motion of interphase chromosomes in budding yeast, limiting chromosome dynamics (Berger et al., 2008; Therizols et al., 2010; Wong et al., 2012; Agmon et al., 2013; Verdaasdonk et al., 2013; Strecker et al., 2016; Lawrimore et al., 2017). Releasing telomere and centromere attachments reproduces chromatin mobility observed in response to DSBs (Strecker et al., 2016; Lawrimore et al., 2017). These studies also identified a Mec1-dependent phosphorylation of the kinetocore protein Cep3 as an essential player in global chromatin mobilization (Strecker et al., 2016). In addition to checkpoint kinases, Rad51 and Rad52 HR proteins are required to facilitate global chromatin dynamics (Seeber et al., 2013; Miné-Hattab et al., 2017; Smith et al., 2018, 2019). Further, studies in yeast and mammalian cells suggest that cytoplasmic actin and microtubules induce a global chromatin “shake-up” in response to DSB formation (Lottersberger et al., 2015; Spichal et al., 2016; Amitai et al., 2017; Lawrimore et al., 2017). Finally, intrinsic modifications of chromatin properties following DSBs, such as chromatin decondensation and changes in chromatin stiffness, appear to contribute to the global increase in chromatin dynamics. Global chromatin decondensation in response to DNA damage has been described across different model systems and likely results from histone modifications, chromatin remodeling, and histone loss (Ziv et al., 2006; Ayoub et al., 2008; Chiolo et al., 2011; Luijsterburg et al., 2012; Seeber et al., 2013; Strecker et al., 2016; Amitai et al., 2017; Hauer et al., 2017). These modifications might promote nuclear exploration by reducing chromatin compaction and increasing its flexibility. Additional studies applied numerical simulation of chromatin dynamics, mainly based on Rouse-like models (Arbona et al., 2017), to predict chromatin mobility in response to DSBs both at the damaged site and genome-wide (Herbert et al., 2017; Lawrimore et al., 2017; Miné-Hattab et al., 2017). For example, β-polymer modeling and simulations suggest that local chromatin expansion is sufficient to drive extrusion of the damage site from its local domain, affecting longer-range dynamics (Amitai et al., 2017). However, multi-scale tracking of chromatin (see: *Multi-scale motion* section, below) and polymer simulations also suggest the importance of chromatin stiffening in local and global chromatin dynamics (Herbert et al., 2017; Lawrimore et al., 2017; Miné-Hattab et al., 2017). This is potentially in contradiction with the role of chromatin relaxation in the same responses, and might reflect a different extent of relaxation/stiffening across distinct loci or time points following damage formation. Thus, more studies are needed to establish the relative contribution of chromatin stiffening and relaxation to increased chromatin exploration, toward an integrated model for damage-induced chromatin dynamics.

Of note, studies in yeast revealed that increased nuclear exploration does not correlate with higher speed of locus movement. In fact, the diffusion coefficient does not significantly change in response to damage, both at damaged and undamaged loci (Miné-Hattab and Rothstein, 2012; Mine-Hattab and Rothstein, 2013). In other words, changes in mobility allow chromatin to go further but not faster.

Overall, MSD analyses have been an invaluable tool for identifying damage-induced nuclear dynamics, revealing a significant increase of nuclear exploration in response to DSBs for both damaged and undamaged chromatin, and identifying several molecular mechanisms responsible for these dynamics.

## Limitations of MSD Analyses

Recent studies of chromatin trajectories in response to DNA damage revealed that MSD analyses also suffer from several limitations, and can even mask the existence of certain characteristics of the motion. First, MSDs are typically calculated as time-ensemble-averaged values over several trajectories to obtain a precise estimate of the parameters describing the motion (e.g., confinement radius and diffusion coefficient). This is in part to compensate for the location measurement errors mentioned above, and in part to enable the use of relatively short trajectories limited by photo-bleaching and photo-toxicity effects. However, averaging the behavior of several trajectories affects the ability to detect the differences between them, i.e., it does not account for heterogeneity across different cells and break sites. Second, MSD calculations assume that each site undergoes homogenous motion during the time of acquisition, which is rarely the case. For example: (i) repair sites can be transiently bound to the nuclear periphery or other nuclear structures; (ii) their motion can be different inside or outside phase separated domains; and (iii) directed motions can occur for limited time periods (Figure 2B, left). A locus can also undergo distinct diffusion regimes at different time scales, which simultaneously contribute to the motion of a particle. For example, a locus can exhibit a subdiffusive motion characterized by A_micro_, in a region that itself moves with a diffusion coefficient A_macro_ (Figure 2B, right). Additionally, chromatin motion is not purely sub-diffusive even in the absence of DNA damage; studies in budding yeast (Heun et al., 2001) and Chinese hamster ovary cells (Levi et al., 2005) showed that chromatin undergoes confined random motion alternating with rare fast jumps that likely reflect rare events of active diffusion.

**FIGURE 3:**
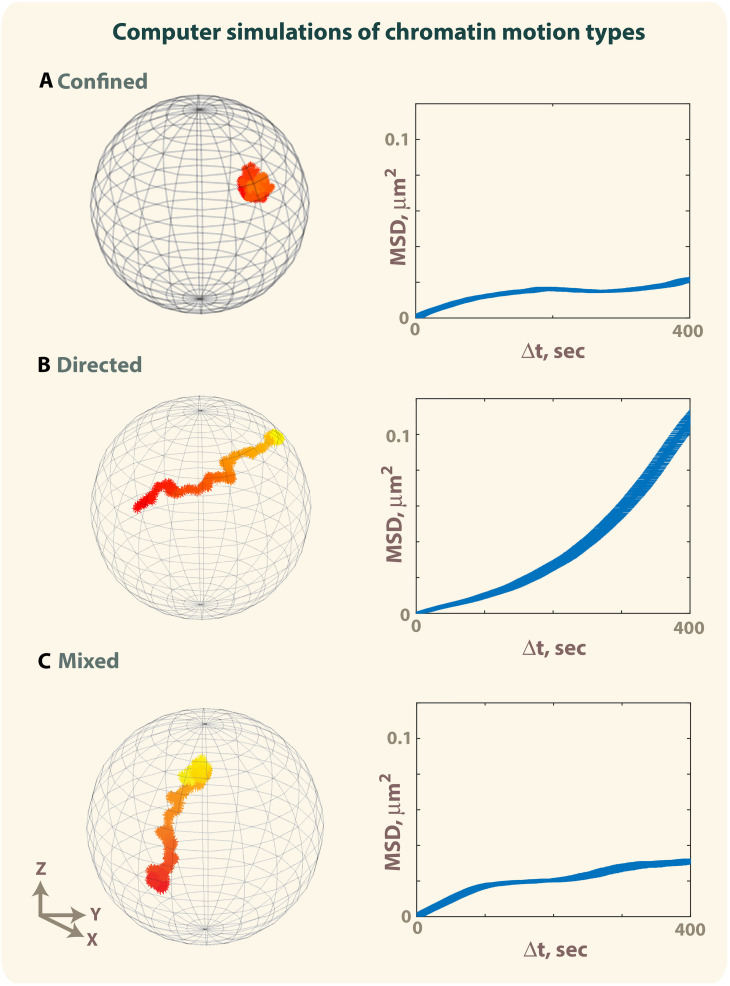
Simulation of confined motion, directed motion and mixed trajectories. The motion of a particle in a sphere of 1 μm radius was simulated using 1,000 iterations (Bacher et al., 2004; Masedunskas et al., 2017). Early timepoints are colored in red, late timepoints in yellow. **(A)** Example of a trajectory obtained by simulating a confined motion (*D* = 0.005 μm^2^/s, Rc = 0.3 μm) (see also corresponding Supplementary Movie S1). **(B)** Example of a trajectory obtained by simulating directed motion until the particle reaches the surface of the sphere (*D* = 0.005 μm^2^/s, ν = 1 μm) (see also corresponding Supplementary Movie S2). **(C)** Example of a mixed trajectory characterized by confined motion (*D* = 0.005 μm^2^/s, Rc = 0.3 μm) lasting 200 timepoints, followed by directed motion (*D* = 0.005 μm^2^/s, ν = 1 μm for *t* = 201–400) and confined motion for the last 600 time points (*D* = 0.005 μm^2^/s, Rc = 0.3 μm. Time-ensemble MSDs were calculated over 10 trajectories. For panel **(C)**, each trajectory is characterized by a different time point when the directed motion starts, and different duration of the directed motion (see also Supplementary Movie S3).

**FIGURE 4:**
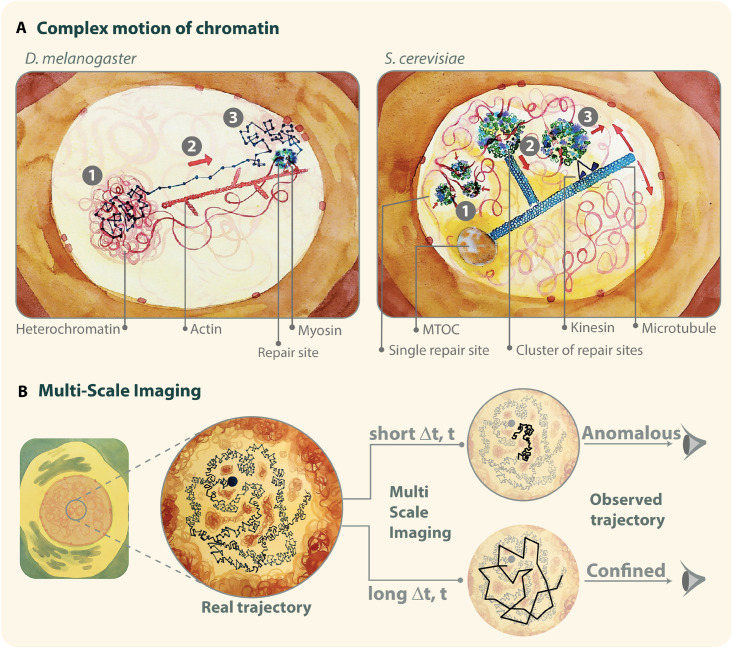
**(A)** Illustration of examples of mixed trajectories. Left: in Drosophila cells, the motion of DSBs leaving the heterochromatin domain and reaching the nuclear periphery is characterized by: (1) confined diffusion inside the heterochromatin domain; (2) myosin-driven directed motion along actin filaments between the heterochromatin domain periphery and the nuclear periphery; and (3) confined diffusion at the nuclear periphery. Right: in budding yeast, repair sites: (1) cluster into larger foci; (2) are “captured” by short microtubules; and (3) move by kinesin-driven directed motions along long nuclear microtubules that pivot around the microtubule organizing center (MTOC). **(B)** Illustration of multi-scale motion. Chromatin imaging at different time scales reveals anomalous diffusion at short time intervals (Δt) and confined diffusion at longer time intervals. It is important to keep in mind that experiments reveal only the mobility of molecules accessible with the specific imaging conditions used during the acquisition. Thus, different imaging settings can shed light on different diffusive behaviors, which are visible only at certain time scales. Illustration by Olga Markova.

Accordingly, simulations of a particle moving with different types of motions: confined, directed, and a combination of confined and directed (mixed trajectory), show how MSD curves can mask the presence of directed motions (Figure 3 and Supplementary Movies S1–S3) (Bacher et al., 2004; Masedunskas et al., 2017). The simulation of a mixed trajectory accounts for asynchronous motion, where the starting point of directed motion and its duration is different for each particle as observed experimentally. While MSD curves for confined and directed motions display the expected shapes (Figures 4A,B), the MSD graph for mixed trajectories resembles that describing a subdiffusive confined motion (Figure 4C), confirming that the MSD approach is not suitable to describe heterogenous and asynchronous motions.

In the following sections, we will illustrate two major types of complex motions occurring in response to DNA damage (Figure 4), and we will discuss experimental approaches and analytical methods that enabled their characterization beyond simple MSD analyses.

## Mixed Trajectories

A major question in the field of nuclear dynamics is whether repair focus motion is driven by active forces, or alternatively subdiffusive motions followed by anchoring to subnuclear structures are sufficient to generate these dynamics. Recent studies revealed the existence of directed motions in a context of mixed trajectories for at least some damage-induced responses (reviewed in Caridi et al., 2019).

First, IR-induced heterochromatic repair foci that relocalize to the nuclear periphery in *Drosophila* cells, are characterized by directed motion driven by transient nuclear actin filaments (F-actin) and myosins (Caridi et al., 2018a; Dialynas et al., 2019; See et al., 2020) (reviewed in Caridi et al., 2019) (Figure 4A, left). Repair foci slide along the filaments, and focus movement requires myosins’ ability to walk along filaments, suggesting that nuclear F-actin provides “highways” for the relocalization of repair sites *via* myosin motors (Caridi et al., 2018a). Myosins and the actin nucleator Arp2/3 associate with the heterochromatin repair component Smc5/6 in response to damage, suggesting Smc5/6 as a physical link between heterochromatic repair sites and the motor system (Caridi et al., 2018a). Smc5/6 also recruits the myosin activator Unc45 to repair sites, inducing chromatin mobilization (Caridi et al., 2018a). Further, relocalization requires SUMOylation, checkpoint and resection proteins, similar to other relocalization pathways (Chiolo et al., 2011; Ryu et al., 2015, 2016; Amaral et al., 2017; Caridi et al., 2018a). Defective relocalization results in unrepaired or misrepaired DSBs, revealing the importance of this pathway for “safe” HR in heterochromatin (Chiolo et al., 2011; Ryu et al., 2015, 2016; Caridi et al., 2018a; Dialynas et al., 2019). Notably, in this context, directed motions primarily occur between the periphery of the heterochromatin domain [a distinct structure in *Drosophila* cells (Chiolo et al., 2011; Li et al., 2017)] and the nuclear periphery, which is where most nuclear actin filaments are organized (Caridi et al., 2018a). Directed motions typically last 24 min, corresponding to the average time required for repair sites to reach the nuclear periphery and the average duration of nuclear actin filaments (Caridi et al., 2018a). However, time points coinciding with the initial movement of repair sites from inside the heterochromatin domain to its periphery are characterized by confined diffusion (Caridi et al., 2018a; Rawal et al., 2019), similar to the rest of undamaged heterochromatin that behaves like a phase separated domain (Larson et al., 2017; Strom et al., 2017). Time points following focus association with the nuclear periphery also display confined diffusion (Ryu et al., 2015; Caridi et al., 2018a; Rawal et al., 2019). In this context where directed motions alternate with diffusive motions, and initiate asynchronously in the population of foci, directed motions are not detected in a simple time-ensemble MSD analysis (Ryu et al., 2015; Caridi et al., 2018a, b) (Supplementary Table S1). Time points characterized by directed motions were identified using an analytical method that scans the trajectory of each focus at variable time windows and initiation times, and detects time windows in which MSD graphs displays upward curvature (Caridi et al., 2018a, b) (Supplementary Table S1). Isolating these time points also required imaging techniques that minimize cell movement and correct for modest rotational and translational motion of the nuclei (Amitai et al., 2017), removing a significant amount of noise from the system (Caridi et al., 2018b; See et al., 2020) (Supplementary Table S1). Additionally, given the long time span along which these motions occur, optimizing imaging conditions for long time imaging and sufficiently spaced time intervals is essential for their detection (Ryu et al., 2015; Caridi et al., 2018a, b; See et al., 2020).

Notably, these studies also established that the average speed of focus motion associated with the relocalization of heterochromatic DSBs is not higher at time points characterized by directed motion relative to time points characterized by confined diffusion (Caridi et al., 2018a; Rawal et al., 2019). This is consistent with a model where actin filaments and motors do not increase motion speed. Rather, they provide directionality and counteract other forces that might limit the release of repair foci from the heterochromatin domain (e.g., chromatin compaction and/or phase separation) (Rawal et al., 2019).

Second, in a study currently in preprint, application of similar analysis methods identified short time points characterized by directed motions for damaged replication forks in human cells, which also correlate with the formation of nuclear actin filaments and the restart of stalled forks (Lamm et al., 2018).

Third, directed motions have been detected during homology search for HR repair of telomeres in ALT human cells (Cho et al., 2014), which might also potentially include C-circles released from telomeres (Henson et al., 2009; Schrank and Gautier, 2019; Zhang et al., 2019). In this case, time-ensemble-average MSD graphs were characterized by α > 1 when calculated at selected time points preceding telomere-telomere association for ALT repair, effectively limiting the analysis to time points when the motion is homogeneous (Supplementary Table S1).

Fourth, directed motions have been described for subtelomeric DSBs repaired by the HR sub-pathway break-induced replication (BIR) in *S. cerevisiae* (Oshidari et al., 2018) (Figure 4A, right). These damage sites move along a single nuclear microtubule but directed motions are not easily detectable using canonical MSD analyses because of two major confounding effects: (i) DSB movement along microtubules is transient; and (ii) microtubules pivot around the microtubule organizing center (MTOC), resulting in non-linear directed motions (Oshidari et al., 2018). In this case, directed motions were identified by directional change distribution (DCD) analysis, which measures changes in the angle of a trajectory and can reveal broader motion profiles by increasing the temporal coarse graining (Oshidari et al., 2018) (reviewed in Oshidari et al., 2019b) (Supplementary Table S1). This study also identified a role for Kar3 in kinesin-dependent directed motions and BIR completion (Oshidari et al., 2018). Notably, loss of Kar3 does not affect the average speed of motion (Oshidari et al., 2018), suggesting that also in this context, filaments and motors have a role in providing directionality to the repair site motion rather than affecting speed. In addition to these functions, short nuclear microtubules have been proposed to generate a flow that facilitates clustering of repair foci, and additional short filaments departing from these clusters promote the capturing of repair centers by the main microtubule (Oshidari et al., 2019a).

Fifth, application of the DCD analysis also identified directed motions for persistent DSBs that move to the nuclear periphery in budding yeast (Oshidari et al., 2018), reverting the previous conclusion that these are characterized by diffusive motion followed by nuclear periphery anchoring (Amitai et al., 2017).

Finally, Arp2/3 and nuclear actin polymerization contributes to repair focus clustering and HR repair in *Drosophila* and mammalian cells (Caridi et al., 2018a; Schrank et al., 2018), and short actin filaments travel with repair foci in human cells (Schrank et al., 2018), suggesting a direct role of these structures in mobilizing damage sites. While directed motions have not been directly investigated in this context, and myosins do not seem to be involved (Caridi et al., 2018a), the requirement of nuclear filaments suggest that directed motions might also contribute to these dynamics (Caridi et al., 2019).

It is worth noting that, in addition to heterochromatin, other membraneless -or phase separated- compartments exist in the nucleus, including nucleoli and repair foci *per se* (Altmeyer et al., 2015; Frottin et al., 2019; Kilic et al., 2019; Min et al., 2019; Singatulina et al., 2019) (reviewed in Mine-Hattab and Taddei, 2019; Rawal et al., 2019), which can affect the dynamics of repair foci at different levels. Phase separation of a nuclear domain might promote diffusion of repair sites inside the domain, while limiting release from the domain due to surface tension (Hyman et al., 2014). Phase separation properties of repair components might also contribute to the clustering of repair foci into larger structures, promoting local dynamics (Altmeyer et al., 2015; Kilic et al., 2019; Oshidari et al., 2019a). Notably, as repair sites move from one domain to another, their motion is likely to change properties exhibiting successive diffusion regimes, which cannot be detected with time-ensemble MSD analyses (Figure 4A, right). In all these cases, dedicated analytical methods should be applied to characterize the diffusion regimes involved.

Further, damage-induced nuclear dynamics can occur in the context of a dynamic nucleus, which adds rotational motion to the system. In yeast, removal of nuclear rotations *via* Latrunculin treatment enabled the identification of modes of diffusion that are otherwise masked by the nuclear rotational movement (Amitai et al., 2017). In mouse and *Drosophila* cells, these rotational movements were corrected by registering the nuclei relative to repair foci prior to tracking repair sites to establish repair locus trajectories (Ryu et al., 2015; Caridi et al., 2018a, b; See et al., 2020) (Supplementary Table S1).

These studies point to the importance of applying dedicated imaging approaches, image processing methods, and analytical tools to identify directed motions. They also suggest that nuclear structures and motors contribute to repositioning repair sites in more situations than initially thought, including where diffusive motions appear to prevail. More studies are needed to identify repair contexts relying on directed movements and the structural/motor components mediating these dynamics, and more methods need to be developed to account for different types of mixed trajectories.

## Multi-Scale Motion

Chromatin presents several levels of organization, which translates into different scales of chromatin mobility (Mine-Hattab and Darzacq, 2018). These different modes of diffusion can be unraveled by imaging the chromatin at different time-scales (Figure 4B and Supplementary Table S1). For example, in the absence of DNA damage, chromatin undergoes anomalous diffusion when observed at short time intervals (10-ms to 1-s) (Maeshima et al., 2010; Weber et al., 2010; Burnecki et al., 2012; Hajjoul et al., 2013; Lucas et al., 2014; Backlund et al., 2015; Amitai et al., 2017; Miné-Hattab et al., 2017). However, at longer time scales, MSD exhibits a plateau characteristic of confined diffusion, consistent with the chromatin remaining confined inside a sub-volume of the nucleus (Marshall et al., 1997; Heun et al., 2001; Maeshima et al., 2010; Masui et al., 2011; Miné-Hattab and Rothstein, 2012; Backlund et al., 2015).

Several recent studies applied multi scale imaging to characterize chromatin mobility in response to DSBs. Increased exploration of the nuclear space is detected in response to I-SceI-induced DSBs when imaging is done at 1.5s or longer time intervals (Dion et al., 2012; Miné-Hattab and Rothstein, 2012). However, remarkably, imaging at 100 ms time intervals or faster reveals lower mobility of the damaged site relative to undamaged conditions (Miné-Hattab et al., 2017). Given that a shorter time scale for data collection investigates chromatin motion on a smaller temporal and spatial scale, the low mobility observed at short time scales reflects reduced local mobility of the cut site (Miné-Hattab et al., 2017). These dynamics can be modeled assuming that chromatin persistence length (a measure of the bending stiffness of a polymer) globally increases (Herbert et al., 2017; Miné-Hattab et al., 2017). At the damaged sites, such response likely results from the recruitment of the repair machinery that increases chromatin stiffness (Mine et al., 2007). Accordingly, reduced local mobility has been associated with resected DNA and requires Rad51 (Saad et al., 2014; Miné-Hattab et al., 2017).

The reduced mobility detected at lower time scales also characterizes undamaged chromatin, consistent with a global increase in chromatin stiffness that spreads beyond the damaged loci (Herbert et al., 2017; Miné-Hattab et al., 2017). This might depend on H2A phosphorylation, which spreads for kilobases to megabases from the cut site (Rogakou et al., 1999), and introduces negative charges into the chromatin (Herbert et al., 2017). As a consequence of a global increase in chromatin stiffness, intrachromosomal loci become more distant and their dynamics change, as observed experimentally in yeast (Herbert et al., 2017; Miné-Hattab et al., 2017).

It has been proposed that increased rigidity of the chromatin facilitates the movement of the cut site through the dense nucleoplasm (Miné-Hattab et al., 2017). In other words, a stiffer chromatin (with more rigidity associated with the break site) would enable resected DNA to navigate through adjacent obstacles more efficiently, thus allowing it to reach farther targets. The stiffer chromatin would act like a needle to help move damaged DNA through the chromatin mesh, likened to a “ball of yarn” (Miné-Hattab et al., 2017). Of note, there is currently no method to directly measure chromatin flexibility in living cells. The two studies referred to here (Herbert et al., 2017; Miné-Hattab et al., 2017) use indirect methods to assess chromatin stiffness, by comparing conformation and dynamics of tagged chromosomal loci with polymer simulation.

These studies emphasize the importance of interrogating different spatiotemporal scales to understand chromatin motions, potentially revealing distinct dynamic processes and regulatory mechanisms. Additionally, more sophisticated and refined mathematical tools are necessary to account for the composite nature of chromatin motion, and for example to distinguish between the local diffusion of a locus in a region that itself moves with a different mode of diffusion.

## Conclusion and Perspectives

A large number of studies in the past decade have shown that DSBs trigger a larger exploration of the nucleus for damaged and undamaged chromatin sites, and this response is conserved from yeast to mammalian cells. Increasing chromatin confinement radius, or changing the nature of its motion, dramatically enhances the ability of a locus to sample neighboring DNA sequences during homology search. In addition to this response, recent studies have shown that chromatin motion is more complex than initially anticipated. Relocalization of repair sites *via* molecular motors typically results in mixed trajectories, where directed motions occur in alternation with subdiffusive regimes. Further, the transient directed movement of repair sites along oscillating structures (e.g., nuclear microtubules), nuclear flows, and phase separation of nuclear domains, add complexity to the trajectories. Additionally, distinct diffusion regimes typically occur at different time scales, likely reflecting different level of chromatin organization. A simple MSD analysis is not adapted for such composite motions, as it assumes a homogenous mode of diffusion during the acquisition. Additionally, time-ensemble MSD analyses mix different type of motions that start asynchronously and occur for different durations. New analytical methods enabled the dissection of some of these dynamics. To reveal the existence of several diffusion regimes, multi-scale tracking, simulations, and mathematical models of complex motions need to be performed. The identification of several contexts where nuclear dynamics is dependent on nuclear actin filaments or microtubules, and characterized by short or long tracts of directed motions, suggests the existence of forces that drive the motion in more situations than initially thought. The development of new dedicated analytical methods started unlocking the door toward a deeper understanding of these dynamics, and the discovery of the molecular mechanisms responsible for their regulation. Nuclear dynamics facilitate DNA repair in different contexts, but nuclear exploration of damaged sequences is also responsible for chromosome rearrangements (Neumann et al., 2012; Roukos et al., 2013; Marcomini et al., 2018). Defects in relocalization pathways also result in genome instability (Torres-Rosell et al., 2007; Chiolo et al., 2011; Ryu et al., 2015, 2016; Su et al., 2015; Caridi et al., 2018a; Dialynas et al., 2019; Aguilera et al., 2020) (reviewed in Caridi et al., 2017; Caridi et al., 2019; Schrank and Gautier, 2019), and establishing the mechanisms responsible for these dynamics is a necessary step to understand how their misregulation contributes to cancer and other genome instability disorders.

## Author Contributions

Both authors equally contributed to this review and approved the submitted version.

## Conflict of Interest

The authors declare that the research was conducted in the absence of any commercial or financial relationships that could be construed as a potential conflict of interest.
