# Supplementary material for: Complex Chromatin Motions for DNA Repair
Source: Front Genet. 2020 Aug 27;11:800. doi: 10.3389/fgene.2020.00800 (PMC7481375; doi:10.3389/fgene.2020.00800)
Supplement: Supplementary file 1 [file Table_1.pdf]

Supplementary Table 1. Factors affecting MSD calculations

**Factors affecting MSD**		**Causes**	**Remedies**	**Ref.**
**Intrinsic imaging errors**	Location Accuracy Errors	Convolution of the sample with the point spread function (PSF)	- Increase number of photons (exposure time)<br>- Use objectives with high N.A.<br>- Account for errors in MSD calculations	*Miné-Hattab et al., 2017;<br>Michalet et al., 2012*
	Motion Blur	Repair foci move during exposure time	- Lower exposure time<br>- Account for errors in MSD calculations	*Miné-Hattab et al., 2017;<br>Michalet et al., 2012*
**Experimental errors**	Cell movement	Cell moves and nucleus rotates inside the cell	- Cell immobilization on imaging surface (i.e., agar plugs, ConA or Fibronectin coating)<br>- Inactivation of the pathways responsible for nuclear movements<br>- Registration of cells relative to ‘fixed’ structures inside the nucleus	*Caridi et al., 2018a,b;<br>See et al., 2020;<br>Amitai et al., 2017;<br>Bystricky et al., 2005;<br>Eckert-Boulet et al., 2011*
**Heterogeneity of motion**	Cell-to-cell variation	Motion is affected by cell cycle phases	- Include live cell cycle markers to distinguish between cell cycle phases, or arrest cells	*Smith et al., 2019;<br>Schrank et al., 2018*
	Mixed trajectories	Foci undergo different types of motion over time	- Analyze individual MSD<br>- Apply analysis methods that identify time points associated with each type of motion	*Cho et al., 2014;<br>Caridi et al., 2018a,b;<br>Lamm et al., 2018;<br>Oshidari et al., 2018;*
	Asynchronous motions	Foci initiate modes of motion asynchronously in the cell population and/or for variable durations		
**Scale of imaging**	Short or long time points of imaging	Different levels of chromatin organization lead to chromatin mobility on different scales	- Image and perform MSD analyses across different time scales	*Bronstein et al., 2009;<br>Miné-Hattab et al., 2017;<br>Herbert et al., 2017*
